# How do polygenic and exposomic risk factors for schizophrenia impact distressing and persisting psychotic experiences during early adolescence? A longitudinal analysis from the ABCD Study

**DOI:** 10.1192/j.eurpsy.2025.325

**Published:** 2025-08-26

**Authors:** M. Di Vincenzo, T. Prachason, G. Sampogna, A. Arias-Magnasco, B. D. Lin, L.-K. Pries, J. van Os, B. P. F. Rutten, R. Barzilay, A. Fiorillo, S. Guloksuz

**Affiliations:** 1 Department of Psychiatry, University of Campania “Luigi Vanvitelli”, Naples, Italy; 2 Department of Psychiatry, Faculty of Medicine Ramathibodi Hospital, Mahidol University, Bangkok, Thailand; 3 Department of Psychiatry and Neuropsychology, School for Mental Health and Neuroscience, Maastricht University Medical Centre, Maastricht; 4 Department of Psychiatry, UMC Utrecht Brain Centre, University Medical Centre Utrecht, Utrecht University, Utrecht, Netherlands; 5 Department of Psychosis Studies, Institute of Psychiatry, Psychology & Neuroscience, King’s College London, London, United Kingdom; 6 Department of Psychiatry, Perelman School of Medicine, University of Pennsylvania; 7 Lifespan Brain Institute of the Children’s Hospital of Philadelphia and Penn Medicine; 8 Department of Child and Adolescent Psychiatry and Behavioral Science, Children’s Hospital of Philadelphia, Philadelphia; 9 Department of Psychiatry, Yale University School of Medicine, New Haven, United States

## Abstract

**Introduction:**

Psychotic experiences (PEs) are subclinical symptoms of psychosis affecting about 10% of children and adolescents. They may cause significant distress and impair daily functioning. Moreover, when persisting over time they are more likely to result in psychotic disorders.

**Objectives:**

This longitudinal study aimed at assessing the effects of polygenic and environmental risk factors for schizophrenia on distressing and persisting PEs in a cohort of adolescents.

**Methods:**

Data were obtained from participants of European ancestry derived from the *Adolescent Brain and Cognitive Development Study,* Release 5.1. Past-month PEs were assessed using the *Prodromal Questionnaire-Brief Child Version.* The primary outcome was distressing PEs at 3-year follow-up. Secondary outcomes included varying levels of persistence of distressing PEs, occurring in 1, 2, 3, or all 4 waves. Polygenic risk score for schizophrenia (PRS-SCZ) was calculated using the continuous shrinkage approach (PRS-cs). The exposome score for schizophrenia (ES-SCZ) was generated by summing up the weighted risk of nine environmental exposures across lifetime: emotional neglect, physical neglect, emotional abuse, physical abuse, sexual abuse, cannabis use, winter birth, hearing impairment, and bullying. Multilevel logistic regression was carried out to test the individual associations of PRS-SCZ and ES-SCZ with the outcomes; the relative excess risk due to interaction was calculated to determine the additive interaction between PRS-SCZ and ES-SCZ on distressing PEs. Main analysis was adjusted for age and sex as covariates; sensitivity analysis also included family income and parental education.

**Results:**

ES-SCZ was significantly associated with 3-year follow-up distressing PEs (OR 1.27 [95% CI 1.14, 1.43], *p*<.001) and lifetime distressing PEs at all degrees of persistence, with an increasing magnitude of association for a higher degree of symptom persistence (≥1 wave: OR 2.77 [95% CI 2.31, 3.31], *p*<.001; ≥2 waves: OR 3.16 [95% CI 2.54, 3.93], *p*<.001; ≥3 waves: OR 3.93 [95% CI 2.86, 5.40], *p*<.001; all 4 waves: OR 3.65 [95% CI 2.34, 5.70], *p*<.001). PRS-SCZ was significantly associated with distressing PEs persisting for more than one (OR 1.29 [95% CI 1.08, 1.53], *p*=.040) or two waves (OR 1.34 [95% CI 1.08, 1.65], *p*=.070) and also additively interacted with ES-SCZ for these outcomes (≥1 wave: RERI 1.26 [95% CI 0.14, 2.38], *p*=.027; ≥2 waves: RERI 1.79 [95% CI 0.35, 3,23] *p*=.015). Sensitivity analysis confirmed all main results.

**Conclusions:**

PRS-SCZ and ES-SCZ showed independent and joint effects on distressing PEs. The more pronounced contribution of ES-SCZ on distressing PEs and its gradient effects on the degree of persistence calls for particular attention to environmental risk factors for schizophrenia on the development and persistence of PEs.

**Disclosure of Interest:**

None Declared

